# Ultrasound‐Guided Stellate Ganglion Block in Fibromyalgia: A Randomized, Single‐Center, Single‐Blind, Sham‐Controlled Trial

**DOI:** 10.1155/prm/4146674

**Published:** 2026-07-30

**Authors:** Yagmur Can Dadakci

**Affiliations:** ^1^ Department of Algology, Sanliurfa Egitim ve Arastirma Hastanesi, 63290, Sanliurfa, Turkey; ^2^ Department of Algology, Sanliurfa Education and Research Hospital, Sanliurfa, Turkey, sueadh.gov.tr

**Keywords:** chronic pain, fibromyalgia, interventional pain management, numerical rating scale, randomized controlled trial, stellate ganglion block, sympathetic nervous system

## Abstract

**Introduction:**

Fibromyalgia is a chronic pain syndrome characterized by widespread pain, fatigue, and sleep disturbance. Many patients remain symptomatic despite guideline‐concordant pharmacologic therapy. Stellate ganglion block (SGB) modulates sympathetic activity and may provide an adjunctive option for fibromyalgia‐related pain.

**Methods:**

In this single‐center, randomized, single‐blind, sham‐controlled trial, adults fulfilling the 2016 American College of Rheumatology criteria and receiving stable duloxetine 60 mg/day were randomized 1:1 to ultrasound‐guided SGB or a sham procedure. Two procedures were performed 1 week apart. The primary endpoint was change in the Fibromyalgia Impact Questionnaire Revised (FIQR) total score from baseline to 1 week. Secondary endpoints included FIQR change at 1 month, pain intensity on an 11‐point Numerical Rating Scale (NRS), and FIQR responder rates (≥ 30% and ≥ 50% improvement). Primary analyses were per‐protocol (*n* = 60), with modified intention‐to‐treat (mITT, *n* = 60) and last observation carried forward (LOCF, *n* = 68) sensitivity analyses. ANCOVA was adjusted for baseline FIQR.

**Results:**

Of 108 screened patients, 68 were randomized (SGB *n* = 33; control *n* = 35); 60 (30 per group) completed all assessments. Baseline FIQR was 87.7 ± 7.1 in the SGB group and 82.0 ± 11.3 in controls. At 1 week, FIQR decreased to 53.4 ± 19.0 with SGB versus 77.2 ± 16.3 with control; at 1 month, FIQR was 63.1 ± 20.3 versus 80.2 ± 10.7, respectively. ANCOVA‐adjusted between‐group differences in FIQR change favored SGB by −29.9 points (95% CI −37.9 to −21.9; *p* < 0.001) at 1 week and −22.9 points (95% CI −30.1 to −15.7; *p* < 0.001) at 1 month. NRS pain reductions were also significantly greater with SGB at both time points (adjusted difference −3.1 points, 95% CI −4.1 to −2.0, *p* < 0.001 at 1 week; −2.6 points, 95% CI −3.6 to −1.6, *p* < 0.001 at 1 month). No serious complications occurred; adverse events were transient and mild.

**Conclusion:**

In treatment‐resistant fibromyalgia patients receiving stable duloxetine, ultrasound‐guided SGB produced clinically meaningful short‐term improvements in fibromyalgia impact and pain with an acceptable safety profile. However, the large effect sizes should be interpreted cautiously given potential unblinding and placebo effects. Larger, multicenter trials with longer follow‐up, objective outcome measures, and formal blinding assessment are warranted.

**Trial Registration:** ClinicalTrials.gov Identifier: NCT07343128

**Plain Language Summary**
• Fibromyalgia is a long‐lasting condition that causes widespread body pain, tiredness, and poor sleep. Many people with fibromyalgia do not feel better enough with current medicines. The stellate ganglion is a group of nerves in the neck that helps control pain signals and blood flow. A stellate ganglion block is an injection near this nerve group that temporarily reduces its activity.
• In this study, adults with fibromyalgia who had not improved sufficiently with usual treatments and were taking a stable dose of duloxetine were randomly divided into two groups. One group received an ultrasound‐guided stellate ganglion block. The other group received a sham procedure that looked similar but did not affect the stellate ganglion. Two procedures were performed on both groups 1 week apart. Fibromyalgia symptoms and pain levels were measured on the day of the first procedure, 1 week after the first procedure on the day of the second procedure, and 3 weeks after the second procedure.
• Patients who received the stellate ganglion block showed greater improvements in overall fibromyalgia symptoms and pain intensity than those who received the sham procedure. About two out of three patients in the stellate ganglion block group achieved a meaningful improvement in their fibromyalgia score after 1 week, and about half still had this level of improvement 1 month after starting treatment. No serious side effects were observed; only temporary and mild side effects were seen.
• These results suggest that the stellate ganglion block may be a helpful additional option for some patients who do not get enough relief from standard treatments. However, a key limitation is that the active treatment caused noticeable physical signs (such as a droopy eyelid) that may have allowed patients to guess which treatment they received, potentially influencing their symptom reporting. Larger and longer‐term studies with better blinding methods and objective measurements are needed to confirm these findings.
 
**Key Summary Points**
• Why carry out this study?
◦ Fibromyalgia is associated with substantial pain, disability, and reduced quality of life, and many patients do not achieve adequate symptom control with guideline‐recommended pharmacologic treatments alone.
◦ Stellate ganglion block (SGB) is an established sympathetic block that may modulate autonomic and inflammatory mechanisms thought to contribute to fibromyalgia; high‐quality randomized, sham‐controlled evidence in this setting is lacking.
◦ We hypothesized that adding ultrasound‐guided SGB to stable duloxetine therapy would provide greater improvement in fibromyalgia impact and pain intensity than a sham control procedure.
• What was learned from the study?
◦ In this single‐center, randomized, single‐blind, sham‐controlled trial, SGB plus duloxetine produced significantly greater improvements in FIQR and pain scores at 1 week and 1 month than sham plus duloxetine, with large standardized effect sizes.
◦ Approximately two‐thirds of patients receiving SGB achieved at least a 30% improvement in FIQR at 1 week, and nearly half maintained this response at 1 month, whereas no responders were observed in the control group at 1 month.
◦ Ultrasound‐guided SGB was well tolerated with no serious complications.
◦ The large effect sizes observed should be interpreted with caution, as potential unblinding due to Horner syndrome and the absence of objective outcome measures are important limitations. These findings are hypothesis‐generating and require confirmation in larger, multicenter trials with formal blinding assessment, objective endpoints, and longer follow‐up.

## 1. Introduction

Fibromyalgia syndrome (FMS) is a chronic disorder characterized by widespread musculoskeletal pain accompanied by cognitive, psychiatric, and somatic symptoms, often triggered or exacerbated by physical and psychological stress. The global prevalence of FMS is estimated at approximately 2.7%, but the true rate is likely higher, and the condition is associated with a substantial loss of work productivity, increased healthcare utilization, and a marked decline in quality of life [1]. Despite various pharmacological and nonpharmacological treatment approaches, symptom control remains inadequate in a significant proportion of patients [1–3].

Current evidence suggests that central sensitization, neuroinflammation, autonomic nervous system dysfunction, and neuroendocrine disturbances contribute to the pathogenesis of FMS. Elevated proinflammatory cytokine levels (e.g., tumor necrosis factor‐*α*, interleukin‐6, and interleukin‐8) and sympathetic nervous system hyperactivity are among the findings supporting these mechanisms [4, 5]. These observations draw attention to potential therapeutic strategies targeting the sympathetic nervous system in FMS.

Stellate ganglion block (SGB) is an interventional technique that blocks sympathetic fibers innervating the head, neck, upper extremities, and upper thoracic region and has long been used in complex regional pain syndrome and other chronic pain conditions [6]. SGB may exert analgesic effects by reducing sympathetic activity, modulating regional blood flow, and influencing neuroinflammatory pathways [4–6]. Emerging evidence also suggests positive effects on psychosocial components relevant to FMS, such as depression, anxiety, sleep disturbance, and cognitive complaints. However, most prior studies in this area are small, noncontrolled, or short‐term, limiting the generalizability of their findings [7].

Bengtsson and Bengtsson reported symptomatic improvement with regional sympathetic blockade in primary fibromyalgia [8], and Nakajima et al. observed improvement in fibromyalgia symptoms following xenon irradiation of the stellate ganglion region [9]. These data support the concept that interventions targeting the stellate ganglion may be beneficial in FMS, but high‐quality randomized, sham‐controlled studies are lacking.

The aim of this randomized, single‐center, single‐blind, sham‐controlled study was to evaluate the efficacy and safety of ultrasound‐guided SGB, in addition to fixed‐dose duloxetine treatment, on the overall impact of fibromyalgia and pain intensity in treatment‐resistant FMS patients. We hypothesized that SGB would provide greater improvement in Fibromyalgia Impact Questionnaire Revised (FIQR) scores and pain intensity compared with a sham injection.

## 2. Methods

### 2.1. Study Design and Ethics

This randomized, single‐center, single‐blind, sham‐controlled trial was approved by the Harran University Faculty of Medicine Ethics Committee (decision no. HRU/25.1.30; 26 May 2025) and conducted in accordance with the Declaration of Helsinki and Good Clinical Practice. All participants provided written informed consent after receiving detailed information about the study procedures.

**FIGURE 1:**
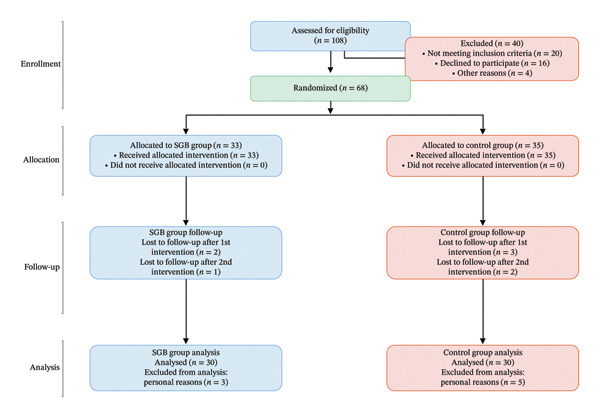
CONSORT flow diagram. Flow diagram showing participant progress through enrollment, allocation, follow‐up, and analysis phases of the randomized trial. Of the 108 patients assessed for eligibility, 68 were randomized to either the stellate ganglion block (SGB) group (*n* = 33) or the control group (*n* = 35). During follow‐up, 3 patients in the SGB group and 5 in the control group were lost to follow‐up or discontinued intervention and were excluded from the per‐protocol analysis. Thus, 60 patients (30 per group) completed all assessments and were included in the final analysis.

Participants were randomized to receive either ultrasound‐guided SGB (interventional treatment) or a sham control procedure. Two procedures (active SGB or sham) were performed 1 week apart. Assessments were conducted at baseline (immediately before the first injection), 1 week after the first injection (on the day of the second procedure), and 3 weeks after the second injection (approximately 1 month after baseline). The flow of participants through the study is summarized in a CONSORT diagram (Figure 1).

### 2.2. Sample Size

The sample size was calculated using the FIQR total score as the primary outcome. A 15‐point between‐group difference in FIQR change at 1 week was considered clinically meaningful [10, 11]. Assuming a standard deviation (SD) of 20 points, a two‐sided *α* of 0.05, and 80% power, 28 patients were required in each group. To account for potential dropouts and protocol deviations, the planned enrollment was 30 patients per group (60 in total).

### 2.3. Participants

Patients aged 18–65 years with a diagnosis of FMS according to the 2016 American College of Rheumatology criteria, followed for at least 1 year, and reporting insufficient benefit from previous pharmacological and/or nonpharmacological treatments were screened. All eligible patients were required to take duloxetine 60 mg once daily for at least 4 weeks prior to inclusion and to continue this fixed dose throughout the study. A fixed daily dose of 60 mg duloxetine was chosen because it represents a commonly used, guideline‐concordant therapeutic dose for fibromyalgia [3], and a stable regimen was required to minimize variability in background pharmacological treatment. Individual dose titration was not permitted during the study to avoid confounding effects on FIQR and Numerical Rating Scale (NRS) outcomes. During follow‐up, no other routine medications for fibromyalgia were administered; however, the use of short‐acting simple analgesics for rescue purposes was permitted.

Exclusion criteria were as follows: nonpharmacological treatments within the previous 6 months (e.g., acupuncture, ozone therapy, or cognitive behavioral therapy); local infection at the injection site; pregnancy or suspected pregnancy; known allergy to local anesthetics; history of malignancy; bleeding or coagulation disorders; use of oral anticoagulants; uncontrolled hypertension, diabetes mellitus, asthma, chronic obstructive pulmonary disease, heart failure, or other significant systemic diseases; psychiatric or cognitive disorders that could interfere with treatment compliance (e.g., severe psychiatric illness or dementia); age < 18 or > 65 years; and refusal of interventional treatment.

Of 108 patients evaluated for eligibility, 68 met the inclusion criteria and were randomized. During follow‐up, outcome data were missing for 3 patients in the SGB group and 5 in the control group. Thus, 60 patients (30 per group) who completed all planned assessments with evaluable FIQR and pain scores were included in the per‐protocol efficacy analyses.

### 2.4. Randomization and Blinding

Patients were randomized in a 1:1 ratio to the SGB group or the control group using a computer‐generated random sequence. To ensure allocation concealment, the randomization sequence and sequentially numbered, opaque, sealed envelopes were prepared by an independent staff member not otherwise involved in the study. The investigator opened the envelopes only at the time of the procedure.

The study employed a single‐blind design, with participants blinded to treatment allocation. To support blinding, patient preparation, positioning, ultrasound imaging, and needle insertion procedures were identical in both groups. Because a single experienced pain physician performed all interventions, operator blinding was not feasible.

To minimize potential performance and detection bias inherent to a single‐investigator design, all primary and secondary efficacy outcomes (FIQR and NRS) were strictly self‐administered by the patients using standardized questionnaires. The investigator provided no guidance or influence during questionnaire completion and remained available only to clarify procedural instructions if required, ensuring that outcome data reflected the patients’ own perceptions of their symptoms. Data entry and statistical analyses were conducted according to a predefined protocol to promote objective reporting.

Stratified randomization based on baseline FIQR or other prognostic variables was not implemented because of the single‐center design, limited sample size, and logistical considerations. Instead, simple randomization was used, and potential baseline imbalances were addressed analytically using analysis of covariance (ANCOVA). Blinding effectiveness was not formally assessed using a postprocedure questionnaire; this is acknowledged as an important limitation (see Limitations).

All procedures were conducted in a dedicated interventional suite, and patients did not have contact with other participants before, during, or after the interventions, thereby minimizing exposure to visual or contextual cues (e.g., observation of Horner syndrome) that could potentially compromise blinding. Participants did not view the ultrasound screen during either procedure due to positioning and screen orientation.

The sham procedure was designed to mimic the active intervention while avoiding sympathetic blockade. Participants were informed that they would receive a SGB and that the procedure involved a neck injection. Patient preparation, positioning, ultrasound probe placement, and skin antisepsis were identical in both groups. In the sham group, a needle was inserted under ultrasound guidance into the sternocleidomastoid muscle; intramuscular needle tip position was confirmed on ultrasound for safety, and 2 mL of normal saline was injected intramuscularly. Importantly, the sham procedure did not reproduce the characteristic physiological effects of successful SGB—namely, ipsilateral Horner syndrome (miosis, ptosis, and anhidrosis), facial warmth, and conjunctival injection—which are perceptible to patients and may have compromised blinding integrity. This represents a fundamental challenge in sham‐controlled SGB trials and is discussed further in the Limitations section.

### 2.5. Outcome Measures

Assessments were performed immediately before the first injection (baseline), 1 week after the first injection, and 3 weeks after the second injection (approximately 1 month after baseline).

At each visit, the FIQR was administered to assess the overall impact of fibromyalgia [10, 11]. Pain intensity over the previous week was evaluated using an 11‐point NRS (0 = *no pain*, 10 = *worst pain imaginable*). The primary outcome was change in the FIQR total score from baseline to 1 week after the first intervention.

Secondary outcomes included the FIQR total score at 1 month and the corresponding change from baseline, NRS scores at 1 week and 1 month, and FIQR response rates defined as patients achieving ≥ 30% and ≥ 50% reductions from baseline. Safety outcomes included procedure‐related adverse events and complications.

All outcomes were patient‐reported subjective measures. No objective physiological assessments—such as heart rate variability, actigraphy‐based sleep metrics, quantitative sensory testing, or tender point assessment—were included. This reliance on subjective endpoints is acknowledged as a limitation, as it increases susceptibility to expectation and placebo effects, particularly in the context of potential unblinding (see Limitations).

Patients who missed the 1‐week or 1‐month visit and therefore had missing FIQR or NRS data were excluded from the per‐protocol efficacy analyses. Additional modified intention‐to‐treat (mITT) and sensitivity analyses were conducted as described below. The mITT analysis included all randomized patients who completed at least one postbaseline assessment, regardless of protocol adherence. This approach was employed to retain the benefits of intention‐to‐treat (ITT) principles while addressing the practical limitations posed by missing outcome data in a small trial setting.

### 2.6. Statistical Analysis

Continuous variables were summarized as mean ± SD or median (interquartile range), as appropriate; categorical variables were summarized as counts and percentages. Between‐group differences in FIQR and NRS scores at follow‐up were assessed using ANCOVA, with the corresponding baseline value entered as a covariate and treatment group (SGB vs. control) as a fixed factor [12, 13]. Estimated marginal means (EMMs) were calculated from ANCOVA models at the grand mean of the baseline covariate to provide baseline‐adjusted group‐specific estimates. Unadjusted between‐group differences in means were also calculated descriptively.

ANCOVA model assumptions were formally tested: Homogeneity of regression slopes was assessed by including a group × baseline interaction term; residual normality was evaluated using the Shapiro–Wilk test; and homogeneity of variance was examined using Levene’s test and the Breusch–Pagan test. Results of assumption testing are reported in the Results section.

For the primary and key secondary continuous outcomes, standardized effect sizes (Cohen’s *d*) for between‐group differences in change from baseline were calculated using the pooled SD of the change scores. FIQR responder rates (≥ 30% and ≥ 50% improvement) were compared using Fisher’s exact test, and absolute risk differences (ARDs) with 95% confidence intervals (CIs) were reported. A two‐sided *p* value < 0.05 was considered statistically significant. Throughout the manuscript, between‐group differences are reported with 95% CIs.

As a prespecified sensitivity analysis, a linear mixed‐effects model (LMM) with random intercepts for patients and fixed effects for group, time, and group × time interaction was fitted to assess the robustness of findings to the analytic approach. This model accounts for the repeated‐measures structure of the data and provides estimates of group × time interaction effects that are directly comparable to the ANCOVA‐derived between‐group differences.

In total, 68 patients were randomized (33 SGB, 35 control). For 60 patients (30 per group), FIQR and NRS outcomes were available at all three time points, forming the basis of the per‐protocol and mITT analyses. Among the remaining 8 randomized patients, all had baseline FIQR and NRS scores, but only 3 (1 SGB, 2 control) had partial postbaseline data (e.g., 1‐week but not 1‐month values), and 5 had no postbaseline FIQR or NRS measurements.

In principle, multiple imputation–based ITT analyses are recommended for handling missing data in randomized trials [14–16]. However, in this small trial, the high proportion of patients with entirely missing postbaseline outcomes among those lost to follow‐up and the limited auxiliary information available to inform imputation models rendered a formal multiple imputation ITT analysis suboptimal. We therefore focused on per‐protocol and mITT analyses in the 60 patients with complete follow‐up data, complemented by sensitivity analyses using a last observation carried forward (LOCF) approach for all 68 randomized patients. This approach imputed missing values by carrying forward the last available observation and was selected due to the limited auxiliary data available for more sophisticated imputation techniques in this small trial. Given the observed baseline imbalance in FIQR scores between groups, ANCOVA models explicitly included baseline FIQR as a covariate when estimating between‐group differences in FIQR outcomes, in line with recommended practice [12].

All analyses were performed using IBM SPSS Statistics for Windows, Version 26.0 (IBM Corp., Armonk, NY, USA) and R Version 4.3.0 (R Foundation for Statistical Computing, Vienna, Austria) for mixed‐effects models.

## 3. Results

### 3.1. Patient Disposition and Baseline Characteristics

A total of 108 patients underwent initial screening, leading to the randomization of 68 participants (33 in the SGB group and 35 in the control group). During the follow‐up period, eight patients were lost due to reasons unrelated to the study interventions. Specifically, in the SGB arm, two patients missed the 1‐week assessment, and one missed the 1‐month visit. Within the control group, three patients were absent at 1 week and two at 1 month. Consequently, 60 patients (30 per group) provided complete data for the per‐protocol analysis. Baseline characteristics for this population are detailed in Table 1. The mean age was 43.3 ± 11.8 years for SGB and 45.7 ± 12.5 years for controls, with a predominant female representation in both cohorts (83.3% and 76.7%, respectively). While baseline NRS pain scores were comparable (9.5 ± 0.8 vs 9.1 ± 1.0; *p* = 0.104), the SGB group exhibited a significantly higher initial disease burden as measured by the FIQR (87.7 ± 7.1 vs 82.0 ± 11.3; *p* = 0.022).

**TABLE 1:** Baseline demographic and clinical characteristics of the study population (per‐protocol and mITT populations, *n* = 60).

Characteristic	SGB (*n* = 30)	Control (*n* = 30)	*p* value
Age, years, mean ± SD	43.3 ± 11.8	45.7 ± 12.5	0.453
Female sex, *n* (%)	25 (83.3%)	23 (76.7%)	0.748
FIQR total score, baseline, mean ± SD	87.7 ± 7.1	82.0 ± 11.3	0.022
NRS pain, baseline, mean ± SD	9.5 ± 0.8	9.1 ± 1.0	0.104

*Note:* Data are presented as mean ± standard deviation (SD) or *n* (%), as appropriate.

Abbreviations: FIQR, Fibromyalgia Impact Questionnaire Revised; mITT, modified intention‐to‐treat; NRS, Numerical Rating Scale; SGB, stellate ganglion block.

### 3.2. ANCOVA Assumption Testing

Prior to the primary ANCOVA analyses, model assumptions were formally evaluated. For the FIQR outcome at 1 week, the group × baseline FIQR interaction was nonsignificant (*p* = 0.789), confirming homogeneity of regression slopes. Levene’s test indicated homogeneity of variance (*p* = 0.452), and the Breusch–Pagan test was nonsignificant (*p* = 0.134). Although the Shapiro–Wilk test for residual normality reached significance (*W* = 0.924, *p* = 0.001), ANCOVA is known to be robust to moderate departures from normality, particularly with balanced group sizes [12]. For the 1‐month FIQR outcome, the homogeneity of slopes assumption was also satisfied (interaction *p* = 0.066), and residuals were normally distributed (Shapiro–Wilk *p* = 0.370). NRS models demonstrated adequate adherence to all ANCOVA assumptions. Given these results, ANCOVA was considered appropriate for the primary analyses.

### 3.3. FIQR Outcomes

**FIGURE 2:**
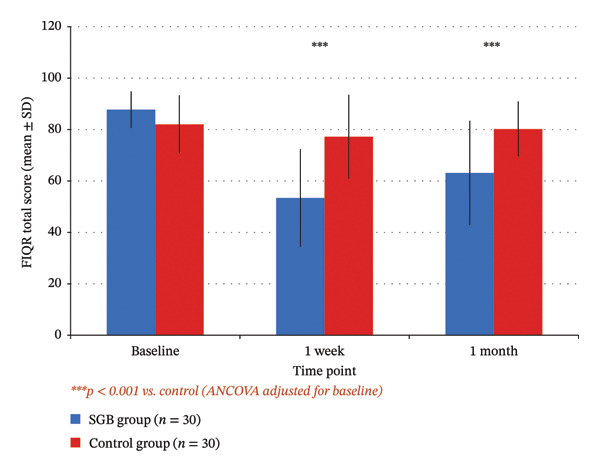
Change in FIQR scores over time. Mean Fibromyalgia Impact Questionnaire Revised (FIQR) scores with standard deviation (SD) are shown for the stellate ganglion block (SGB) and control groups at baseline, 1 week, and 1 month. The SGB group demonstrated a significant reduction in FIQR scores compared to the control group at both follow‐up time points. ^∗∗∗^ *p* < 0.001 vs. control (ANCOVA adjusted for baseline).

The longitudinal progression of FIQR scores is presented in Table 2 and Figure 2. By the first week, mean FIQR scores declined to 53.4 ± 19.0 in the SGB group compared to 77.2 ± 16.3 in the control group. At the 1‐month mark, these values were 63.1 ± 20.3 and 80.2 ± 10.7, respectively. Utilizing ANCOVA to adjust for baseline imbalances, the EMMs (at the grand mean baseline FIQR of 84.8) were 50.4 for SGB and 80.3 for control at 1 week, and 60.2 for SGB and 83.1 for control at 1 month. The adjusted between‐group difference favored SGB by −29.9 points (95% CI −37.9 to −21.9; *p* < 0.001) at 1 week and −22.9 points (95% CI −30.1 to −15.7; *p* < 0.001) at 1 month. These differences corresponded to substantial standardized effect sizes (Cohen’s *d* = 2.01 at 1 week; *d* = 1.73 at 1 month). Sensitivity analyses using the LOCF method for the full randomized sample (*n* = 68) yielded consistent results, reinforcing the primary findings.

**TABLE 2:** FIQR scores at baseline and follow‐up visits with estimated marginal means (per‐protocol population, *n* = 60).

Time point	SGB (*n* = 30)	Control (*n* = 30)	Adjusted difference (95% CI)	*p* value
	Mean ± SD	Mean ± SD		
Baseline	87.7 ± 7.1	82.0 ± 11.3	—	0.022
1 week	53.4 ± 19.0	77.2 ± 16.3	−29.9 (−37.9 to −21.9)	< 0.001
1 month	63.1 ± 20.3	80.2 ± 10.7	−22.9 (−30.1 to −15.7)	< 0.001
Estimated marginal means (at grand mean baseline FIQR = 84.8)				
1 week (EMM)	50.4	80.3	−29.9 (−37.9 to −21.9)	< 0.001
1 month (EMM)	60.2	83.1	−22.9 (−30.1 to −15.7)	< 0.001

*Note:* Data are presented as mean ± standard deviation (SD). Adjusted differences were calculated using ANCOVA with baseline FIQR as covariate.

Abbreviations: CI, confidence interval; EMM, estimated marginal mean; FIQR, Fibromyalgia Impact Questionnaire Revised; SGB, stellate ganglion block.

### 3.4. Pain Intensity Outcomes

**FIGURE 3:**
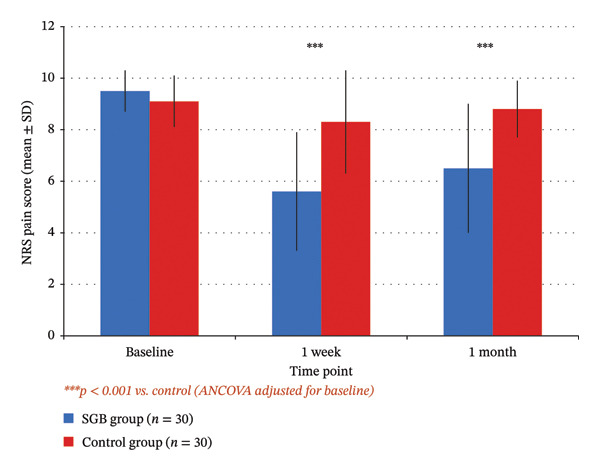
Change in NRS pain scores over time. Mean Numerical Rating Scale (NRS) pain scores with standard deviation (SD) are shown for the stellate ganglion block (SGB) and control groups at baseline, 1 week, and 1 month. The SGB group showed significantly greater reductions in pain intensity compared to the control group at both follow‐up time points. ^∗∗∗^ *p* < 0.001 vs. control (ANCOVA adjusted for baseline).

NRS pain scores followed a similar downward trend in the SGB group (Table 3, Figure 3). At 1 week, scores decreased to 5.6 ± 2.3 with SGB versus 8.3 ± 2.0 in the control arm. At 1 month, the scores were 6.5 ± 2.5 and 8.8 ± 1.1, respectively. The ANCOVA‐adjusted EMMs (at the grand mean baseline NRS of 9.3) were 5.4 for SGB and 8.5 for control at 1 week, and 6.3 for SGB and 8.9 for control at 1 month. Adjusted between‐group differences significantly favored SGB: −3.1 points (95% CI −4.1 to −2.0; *p* < 0.001) at 1 week and −2.6 points (95% CI −3.6 to −1.6; *p* < 0.001) at 1 month, with large effect sizes (Cohen’s *d* = 1.57 at 1 week and 1.46 at 1 month).

**TABLE 3:** NRS pain scores at baseline and follow‐up visits with estimated marginal means (per‐protocol population, *n* = 60).

Time point	SGB (*n* = 30)	Control (*n* = 30)	Adjusted difference (95% CI)	*p* value
	Mean ± SD	Mean ± SD		
Baseline	9.5 ± 0.8	9.1 ± 1.0	—	0.104
1 week	5.6 ± 2.3	8.3 ± 2.0	−3.1 (−4.1 to −2.0)	< 0.001
1 month	6.5 ± 2.5	8.8 ± 1.1	−2.6 (−3.6 to −1.6)	< 0.001
Estimated marginal means (at grand mean baseline NRS = 9.3)				
1 week (EMM)	5.4	8.5	−3.1 (−4.1 to −2.0)	< 0.001
1 month (EMM)	6.3	8.9	−2.6 (−3.6 to −1.6)	< 0.001

*Note:* Data are presented as mean ± standard deviation (SD). Adjusted differences were calculated using ANCOVA with baseline NRS as covariate.

Abbreviations: CI, confidence interval; EMM, estimated marginal mean; NRS, Numerical Rating Scale; SGB, stellate ganglion block.

### 3.5. LMM Sensitivity Analysis

To further assess the robustness of findings, a LMM with random intercepts for patients confirmed significant group × time interactions for both FIQR and NRS outcomes. For the FIQR, the group × time interaction effects were −29.6 (95% CI −36.4 to −22.7; *p* < 0.001) at 1 week and −22.8 (95% CI −29.6 to −16.0; *p* < 0.001) at 1 month, closely matching the ANCOVA‐derived estimates. For the NRS, interaction effects were −3.1 (95% CI −4.0 to −2.2; *p* < 0.001) at 1 week and −2.7 (95% CI −3.7 to −1.8; *p* < 0.001) at 1 month. The consistency of results across ANCOVA, mITT, LOCF, and mixed‐effects model approaches supports the robustness of the observed treatment effect. Changes from baseline in FIQR and NRS scores in the mITT population are presented in Table 4.

**TABLE 4:** Change from baseline in FIQR and NRS pain scores (modified intention‐to‐treat population, *n* = 60).

Outcome	SGB (*n* = 30)	Control (*n* = 30)	Between‐group difference	95% CI	*p* value
	Mean ± SD	Mean ± SD			
FIQR change at 1 week	−34.3 ± 17.2	−4.7 ± 11.5	−29.5	−37.0 to −22.1	< 0.001
FIQR change at 1 month	−24.6 ± 17.5	−1.8 ± 6.2	−22.8	−29.4 to −16.1	< 0.001
NRS pain change at 1 week	−3.9 ± 2.3	−0.8 ± 1.6	−3.1	−4.1 to −2.1	< 0.001
NRS pain change at 1 month	−3.1 ± 2.5	−0.3 ± 0.8	−2.7	−3.7 to −1.8	< 0.001

*Note:* Data are presented as mean ± standard deviation (SD). Negative values indicate improvement (reduction in score or pain). Between‐group differences were calculated as SGB minus control.

Abbreviations: CI, confidence interval; FIQR, Fibromyalgia Impact Questionnaire Revised; NRS, Numerical Rating Scale; SGB, stellate ganglion block.

### 3.6. FIQR Responder Analyses

Responder rates, defined by clinically meaningful thresholds, are summarized in Table 5. At 1 week, 66.7% (*n* = 20) of the SGB group achieved *a* ≥ 30% FIQR improvement, compared to only 6.7% (*n* = 2) in the control group (ARD 0.60; 95% CI 0.41–0.79; *p* < 0.001). This translates to a number needed to treat (NNT) of approximately 2. At 1 month, 46.7% of SGB patients maintained this response level, while no responders were identified in the control group (ARD 0.47; 95% CI 0.29–0.65; *p* < 0.001). Similar significant trends were observed for the more stringent ≥ 50% improvement threshold.

**TABLE 5:** FIQR responder rates at 1 week and 1 month (modified intention‐to‐treat population, *n* = 60).

Time point	Responder definition	SGB (*n* = 30)	Control (*n* = 30)	ARD (SGB – control)	95% CI
		*n* (%)	*n* (%)		
1 week	≥ 30% FIQR	20 (66.7%)	2 (6.7%)	0.60	0.41 to 0.79
1 week	≥ 50% FIQR	9 (30.0%)	0 (0.0%)	0.30	0.14 to 0.46
1 month	≥ 30% FIQR	14 (46.7%)	0 (0.0%)	0.47	0.29 to 0.65
1 month	≥ 50% FIQR	6 (20.0%)	0 (0.0%)	0.20	0.06 to 0.34

*Note:* Data are presented as *n* (%). All comparisons *p* < 0.001 (Fisher’s exact test, two‐sided).

Abbreviations: ARD, absolute risk difference; CI, confidence interval; FIQR, Fibromyalgia Impact Questionnaire Revised; SGB, stellate ganglion block.

### 3.7. Safety

The procedure was generally well tolerated, and no serious complications were recorded. Ipsilateral Horner syndrome was observed in all SGB participants, serving as a clinical indicator of successful sympathetic blockade. Reported adverse events were transient and mild, including hoarseness, dysphagia, and localized injection‐site discomfort, all of which resolved in a short time. No cardiovascular or respiratory events occurred, and no persistent neurological deficits were noted. While the use of short‐acting simple analgesics for rescue was permitted, no patients spontaneously reported frequent analgesic use during the follow‐up period, although rescue medication consumption was not systematically tracked.

## 4. Discussion

In this prospective, randomized, single‐blind, sham‐controlled, single‐center trial, ultrasound‐guided SGB in treatment‐resistant FMS patients receiving stable duloxetine therapy provided statistically and clinically significant short‐term improvements in the overall impact of FMS and in pain intensity compared with a sham procedure.

Compared with the control group, SGB treatment resulted in substantially greater reductions in FIQR and NRS scores at 1 week, with benefits that persisted, although attenuated, at 1 month. Responder analyses showed that approximately two‐thirds of SGB patients achieved at least a 30% improvement in FIQR at 1 week, and nearly half maintained this level of improvement at 1 month, whereas no responders were observed in the control group at 1 month. The magnitude of these effects, with Cohen’s *d* values exceeding 1.5 for key outcomes, is large for an interventional study in chronic pain and suggests a robust treatment signal.

These findings support the hypothesized role of sympathetic nervous system hyperactivity and neuroinflammation in FMS pathophysiology [1, 4, 5, 17–21]. SGB is a well‐established technique in conditions in which sympathetic activity contributes to pain and dysautonomia [6, 22–24]. Sympathetic blockade achieved by SGB may influence autonomic tone, regional blood flow, and inflammatory pathways, and these combined effects may improve fibromyalgia symptoms [4–6, 17–21]. Our results add to the limited existing evidence by demonstrating clinically meaningful benefits in patients who remained symptomatic despite optimized duloxetine therapy.

Although randomization should, in principle, balance prognostic variables, baseline FIQR scores were significantly higher in the SGB group than in the control group, indicating a somewhat greater initial disease burden in the SGB arm. This imbalance would be expected to bias against detecting a benefit of SGB. We addressed it analytically by using ANCOVA with baseline FIQR as a covariate in all models examining FIQR outcomes, as recommended for randomized trials with baseline and follow‐up measurements. ANCOVA assumption testing confirmed the validity of this approach: Homogeneity of regression slopes was satisfied (interaction *p* = 0.789 at 1 week, *p* = 0.066 at 1 month), and variance homogeneity was adequate. The baseline‐adjusted EMMs further confirmed that differences favoring SGB persisted after accounting for the initial imbalance. The consistency of results across ANCOVA, mixed‐effects models, and LOCF sensitivity analyses provides additional reassurance. Nevertheless, residual confounding cannot be entirely excluded, and the baseline imbalance—while unlikely to fully explain the observed effects—highlights the inherent instability of simple randomization in small trials.

### 4.1. Contextualizing Effect Sizes

The remarkably large effect sizes observed in this trial (Cohen’s *d* > 1.5) should be interpreted with particular caution and placed in the context of the broader chronic pain literature. For comparison, pharmacological trials of duloxetine for fibromyalgia typically report effect sizes of *d* ≈ 0.3–0.5 [1, 3]; exercise‐based interventions for chronic pain generally achieve *d* ≈ 0.4–0.8; and most interventional pain procedures rarely exceed *d* ≈ 1.0. Several factors may have contributed to the unusually large effects observed here. First, the highly selected, treatment‐resistant population may have had greater capacity for improvement following a novel intervention. Second, the administration of two SGB procedures may have amplified the treatment effect. Third, the use of standardized stable duloxetine cotherapy provided a uniform background against which SGB effects were measured. Fourth, the relatively short follow‐up period may capture acute procedural effects and regression to the mean, as patients with very high baseline scores are statistically more likely to improve. Fifth, and critically, potential unblinding due to Horner syndrome may have introduced expectation bias that inflated patient‐reported outcomes (discussed below). Accordingly, the true effectiveness of SGB in routine clinical practice is likely to be more modest than suggested by the present point estimates, and these findings should be considered hypothesis‐generating rather than definitive.

### 4.2. Blinding Integrity and Sham Procedure Limitations

A critical limitation of this study is the potential compromise of participant blinding. Ipsilateral Horner syndrome—manifesting as miosis, ptosis, and anhidrosis—was observed in all patients who received active SGB. These are highly recognizable, perceptible signs that patients may have noticed, particularly after repeated procedures. Since the sham procedure (intramuscular saline injection into the sternocleidomastoid) did not reproduce these characteristic physiological effects—including Horner syndrome, facial warmth, vasodilation, and conjunctival injection—patients in the SGB group may have deduced their treatment allocation. This potential unblinding is of particular concern because all primary and secondary outcomes were entirely patient‐reported subjective measures (FIQR and NRS), which are highly susceptible to expectation and placebo effects. In essence, the study may have compared active SGB against a procedure that patients could identify as inactive, rather than a truly credible sham. The absence of a formal blinding assessment (e.g., asking patients “Which treatment do you believe you received?” and “How confident are you?”) represents a significant methodological shortcoming that prevents quantification of the extent of unblinding. This limitation, combined with the exclusive reliance on subjective outcomes, means that the observed treatment effects may be partly attributable to expectation bias. Future trials should incorporate formal blinding questionnaires and, where feasible, explore sham procedures that more closely mimic the physiological responses of active SGB.

The choice of intramuscular saline injection into the sternocleidomastoid muscle as the sham procedure was intended to preserve the visual and sensory aspects of the intervention (ultrasound and needle insertion) without affecting the stellate ganglion itself. While such an approach might exert nonspecific placebo or counterirritant effects, these would be expected to reduce, rather than exaggerate, apparent treatment differences. The absence of FIQR responders in the control group at 1 month likely reflects the high baseline disease burden and treatment resistance of the enrolled population, the absence of concomitant treatment intensification during the trial, and the limited nonspecific effect of the sham intervention in this setting.

Our safety observations are consistent with previous reports on ultrasound‐guided SGB [25–27]. No serious complications occurred, and expected signs of sympathetic blockade such as transient Horner syndrome were universal in the SGB group. These data support the feasibility and short‐term safety of ultrasound‐guided SGB when performed by experienced clinicians under imaging guidance. Nevertheless, SGB requires appropriate emergency preparedness and should be undertaken only in settings with the necessary expertise and facilities.

Our results extend the limited prior literature on stellate ganglion interventions in fibromyalgia. Bengtsson and Bengtsson reported improvements in pain and tenderness after regional sympathetic blockade in primary fibromyalgia; however, only 28 patients were included, SGB was applied to 8 of them, the follow‐up was short (4 h), and tender point counts rather than FIQR were assessed [8]. Nakajima et al. described a series of 27 patients treated with xenon irradiation of the stellate ganglion region in an uncontrolled design [9]. More broadly, data from other populations suggest that SGB may positively influence depression, anxiety, sleep, and cognitive complaints, which are relevant components of FMS [7, 22–24]. Using a randomized, sham‐controlled, single‐blind design and standardized concomitant duloxetine therapy, our study provides more rigorous evidence for a beneficial role of SGB in this context.

## 5. Limitations

This study has several important limitations that should be carefully considered when interpreting the findings.

First, the single‐center, single‐investigator design may increase the risk of performance and detection bias and may limit generalizability. We sought to mitigate this risk by using validated, patient‐reported outcome measures (FIQR and NRS) as primary and secondary endpoints, ensuring that the reported effects reflect patients’ own perceptions rather than investigator assessments. In addition, allocation concealment and predefined analytical protocols were strictly applied to minimize potential bias.

Second, and most critically, blinding integrity is questionable. Ipsilateral Horner syndrome was universally observed in SGB recipients and is a highly recognizable indicator of successful sympathetic blockade. Because the sham procedure did not reproduce the characteristic physiological effects of SGB (Horner syndrome, facial warmth, vasodilation, and ocular symptoms), most patients in the SGB group likely became aware that they had received the active treatment. Since all outcomes were entirely patient‐reported and subjective, the risk of expectation‐driven bias inflating treatment effects is substantial. A formal blinding assessment using a postprocedure questionnaire (e.g., “Which treatment do you believe you received?” and “How confident are you?”) was not performed, and its absence is a significant methodological limitation. Future trials must incorporate such assessments to quantify unblinding and its potential impact on outcomes.

Third, all primary and secondary outcomes relied exclusively on subjective, patient‐reported measures. No objective physiological assessments—such as heart rate variability (to assess autonomic function), actigraphy‐based sleep metrics, quantitative sensory testing, tender point assessment, or activity monitoring—were included. This limits mechanistic interpretation of the findings, as it is not possible to determine whether SGB produced measurable changes in autonomic function or pain processing beyond patients’ self‐reported perceptions. Future studies should incorporate objective outcome measures alongside patient‐reported endpoints to provide a more comprehensive assessment of treatment effects and to support mechanistic hypotheses.

Fourth, the sample size was relatively small, and follow‐up was limited to 1 month after the initial intervention. Small trials are inherently susceptible to baseline imbalances despite randomization, and the observed FIQR imbalance in the present study illustrates this limitation. Consequently, the durability of benefit and the optimal frequency or need for repeated SGB procedures could not be assessed. Larger studies with longer follow‐up are required to evaluate long‐term efficacy and sustainability.

Fifth, despite randomization, baseline FIQR scores were higher in the SGB group than in the control group, indicating a greater initial disease burden in the intervention arm. Although this imbalance would be expected to bias against detecting a treatment effect, we addressed it analytically using baseline‐adjusted ANCOVA, in line with recommended practice. The validity of this approach was supported by formal assumption testing, including nonsignificant tests for homogeneity of regression slopes (*p* = 0.789 at 1 week, *p* = 0.066 at 1 month), adequate residual normality, and homogeneity of variance. Sensitivity analyses using LMM yielded virtually identical estimates, further supporting the robustness of findings. Nevertheless, residual confounding cannot be entirely excluded.

Sixth, although both per‐protocol and mITT analyses were performed, the mITT analysis was limited to participants with at least one postbaseline assessment (*n* = 60). Among the 8 randomized patients lost to follow‐up, only 3 had partial postbaseline data, and 5 had no postbaseline measurements. The absence of a full multiple imputation ITT analysis therefore represents a limitation, and future trials should be prospectively designed to allow more comprehensive handling of missing data.

Seventh, although short‐acting simple analgesics were permitted as rescue medication, their use was not systematically recorded or quantified. This is an important limitation, as differential rescue analgesic use between groups could confound pain outcomes. Even occasional use may influence NRS and FIQR scores, and the inability to account for this represents a potential source of bias. Future trials should systematically record and report rescue medication consumption as a secondary outcome.

Eighth, the extraordinarily large effect sizes (Cohen’s *d* > 1.5) should be interpreted with caution. As discussed above, regression to the mean, potential unblinding, expectation bias, small‐study effects, and the short follow‐up period may all contribute to inflating the apparent treatment benefit. The true effectiveness of SGB in routine clinical practice is likely to be more modest.

Finally, the retrospective nature of the clinical trial registration (registered in January 2026 after enrollment began in June 2025) is a limitation. While the study followed the predefined protocol approved by the ethics committee, prospective registration is the gold standard for ensuring transparency. All procedures were performed by a single experienced operator under ultrasound guidance, which may limit external validity to settings with different operator expertise or access to imaging. Future studies should ensure prospective registration to further validate these findings.

## 6. Conclusion

This randomized, sham‐controlled trial suggests that integrating ultrasound‐guided SGB into a stable duloxetine regimen may offer substantial clinical benefits for patients with treatment‐resistant FMS. The intervention resulted in significant short‐term reductions in both overall fibromyalgia impact and pain intensity when compared to a sham procedure. Furthermore, the technique proved to be safe and well tolerated, with no serious complications reported among the participants.

However, these findings must be interpreted cautiously in light of several important methodological limitations, particularly the potential compromise of blinding due to Horner syndrome, the exclusive reliance on subjective outcomes, and the unusually large effect sizes that may partly reflect expectation bias rather than a true pharmacological effect. These results should therefore be viewed as hypothesis‐generating, providing a foundation for further investigation rather than definitive evidence of efficacy.

To establish the true magnitude of SGB’s therapeutic benefit and the durability of these effects, larger‐scale multicenter trials with extended follow‐up periods, formal blinding assessments, objective outcome measures, systematic rescue medication tracking, and prospective trial registration are essential.

## Author Contributions

Yagmur Can Dadakci was responsible for the study conception and design, performed the clinical interventions, supervised data collection, performed the statistical analysis and data interpretation, and drafted and revised the manuscript. Yagmur Can Dadakci takes full responsibility for the integrity of the data and the accuracy of the data analysis.

## Funding

No funding or sponsorship was received for this study or for the publication of this article.

## Disclosure

*Prior Presentation*: This work has not been previously published and has not been presented in part or in full at any scientific meeting.

*Authorship*: The author meets the International Committee of Medical Journal Editors (ICMJE) criteria for authorship, takes responsibility for the integrity of the work as a whole, and has given final approval for the version to be published.

## Ethics Statement

The study protocol was reviewed and approved by the Harran University Faculty of Medicine Ethics Committee (decision no. HRU/25.1.30, 26 May 2025). The study was conducted in accordance with the principles of the Declaration of Helsinki and Good Clinical Practice. All participants provided written informed consent before enrollment, including consent to publication of anonymized data. Ethical approval was obtained on May 26, 2025, and patient enrollment commenced in June 2025. The trial was registered retrospectively on January 6, 2026.

## Conflicts of Interest

The author declares no conflicts of interest.

## Data Availability

The datasets generated and/or analyzed during the current study are not publicly available because they contain information that could compromise participant privacy, but deidentified data may be made available from the corresponding author upon reasonable request and with appropriate institutional approvals.
